# Unraveling neural coding of dynamic natural visual scenes via convolutional recurrent neural networks

**DOI:** 10.1016/j.patter.2021.100350

**Published:** 2021-09-17

**Authors:** Yajing Zheng, Shanshan Jia, Zhaofei Yu, Jian K. Liu, Tiejun Huang

**Affiliations:** 1 Department of Computer Science and Technology, National Engineering Laboratory for Video Technology, Peking University, Beijing 100871, China; 2 Institute for Artificial Intelligence, Peking University, Beijing 100871, China; 3 School of Computing, University of Leeds, Leeds LS2 9JT, UK

**Keywords:** convolutional neural network, recurrent neural network, neural coding, visual coding, retina, video analysis

## Abstract

Traditional models of retinal system identification analyze the neural response to artificial stimuli using models consisting of predefined components. The model design is limited to prior knowledge, and the artificial stimuli are too simple to be compared with stimuli processed by the retina. To fill in this gap with an explainable model that reveals how a population of neurons work together to encode the larger field of natural scenes, here we used a deep-learning model for identifying the computational elements of the retinal circuit that contribute to learning the dynamics of natural scenes. Experimental results verify that the recurrent connection plays a key role in encoding complex dynamic visual scenes while learning biological computational underpinnings of the retinal circuit. In addition, the proposed models reveal both the shapes and the locations of the spatiotemporal receptive fields of ganglion cells.

## Highlights

• Learning relationship between retinal response and complex visual scenes
• We evaluate quantitatively the complexity of stimuli and spatiotemporal regularity of RFs
• The proposed CRNN can reveal the shapes and locations of receptive fields of RGCs
• The proposed CRNN outperforms CNNs in predicting large populations of RGCs

## The bigger picture

Understanding surrounding environments perceived by eyes requires unraveling the computational principle embedded in the neural system. Recently, deep learning has been implemented to develop useful models of the visual system for studying simple and static scenes. Yet, we perceive continuous dynamic scenes in an ever-changing environment, which cannot be captured by standard convolutional neural networks (CNNs). Here, we use the retina as a model system to demonstrate how recurrence helps to explain the relationship between neural response and complex natural scenes. Leveraging CNNs with different types of recurrence, we highlight the role of recurrence in the neural coding of dynamic visual scenes, not only better predicting the neural response, but also revealing the corresponding biological counterparts. Our results shed new light on unraveling the coding principle of visual neurons for dynamic scenes and provide a way of using recurrence for understanding visual computing.

## Teaser

Visual neuroscience is an immensely popular topic in AI, such that numerous methodologies developed in visual computing have broad applications and provide inspiration for other domains. The retina is one of the best-understood examples in neuroscience for visual computing. Here, we use retinal data to demonstrate how to use deep-learning models to encode dynamic visual scenes. The proposed models demonstrate that recurrence plays a critical role in encoding complex natural scenes and learning the biological computational underpinning of the neural circuits.

## Introduction

Unraveling the neural system of the brain is one of the key questions of both neuroscience and artificial intelligence, as understanding the structure of neural systems could help to develop novel methodologies of artificial intelligence. The visual system constantly receives highly complex and dynamic visual scenes with a high order of spatiotemporal correlations. To cope with these inputs, it is necessary to develop an explainable neural network model, either for explaining the data of neuroscience, e.g., the neural response to input scenes,^1^ or for developing an efficient computational framework for analyzing dynamic visual scenes for artificial vision.^2^

The retina, as the first stage of the visual system, encodes visual information from the external environment in both spatial and temporal domains.^1^^,^^3^ It consists of three layers of neurons, namely, excitatory photoreceptors (input), bipolar cells, and ganglion cells (output), with inhibitory horizontal and amacrine cells communicating within the bipolar and ganglion cell layers, respectively. At the output side of the retina, i.e., the retinal ganglion cells (RGCs), all input signals are transformed into a sequence of spikes. These spikes are then transmitted via the optic nerve to the visual processing center of the brain. The retina receives approximately 100 MB per second of visual input^4^ and sends approximately 1 MB per second of visual data to the brain from 10^6^ RGCs.^5^ Therefore, the retina must be “smart” enough to efficiently encode the input stimuli.^1^ Exploring the encoding mechanism of the retina is essential to unravel the computational principles of other visual systems.

Recent achievements in deep learning have led to renewed interest among researchers using convolutional neural networks (CNNs) to investigate topics in systems neuroscience.^6^, ^7^, ^8^ CNNs have been used to build the most quantitatively accurate models in predicting neural responses.^9^, ^10^, ^11^, ^12^, ^13^ In addition, deep-learning-based methods^14^, ^15^, ^16^, ^17^ have been proposed to model retinal systems and have made remarkable progress in analyzing visual scenes, including those composed of artificial stimuli (e.g., moving bars) and static natural images. These studies have revealed that novel functional neural networks can encode simple and static visual scenes by analyzing the patterns of dynamic responses of RGCs. However, modeling the retina to process dynamics of rather complex natural scenes by deep neural networks remains unclear.^18^

Studies on models of the visual cortex have highlighted the role of recurrent connections in visual processing^19^, ^20^, ^21^ within the models themselves. These connections help “fill in” missing data,^22^, ^23^, ^24^, ^25^ indicating that the real visual cortex allows the brain to “predict” future stimuli.^26^, ^27^, ^28^ In addition, the retina, known as an efficient encoder, can anticipate motion with recurrent connections.^29^ The RGCs can be connected laterally by electric synapses, i.e., gap junctions^30^, ^31^, ^32^, ^33^ or specific amacrine cells. The lateral connection allows the retina to detect the differential motion of the object and background,^34^ while specific asymmetric connectivity of the amacrine cells helps the RGCs show direction selectivity.^35^ These characteristics of gap junctions and recurrent connections play a critical role in the efficient encoding of dynamic visual scenes by the retina.^36^^,^^37^

Therefore, recurrent connections can be a potential element for understanding the neuronal encoding of visual scenes in the retina, which is beyond the capability of the feedforward approach.^14^^,^^15^ The disadvantage of the CNN is that the final fully connected layer maps the convolutional feature space to individual cells' responses, leading to a dramatic increase in the number of model parameters with the increase in the number of neuron inputs. In addition, the CNN models of retinal encoding^14^, ^15^, ^16^ typically learn only the relationship between a stimulus covering a small field of view and the subsequent response of the RGCs. Traditional models for learning retinal coding, such as the generalized linear models,^38^ incorporate several linear or nonlinear filters that model each neuron and a set of coupling filters that capture the neurons' dependencies in the recent activity of other cells. This type of model is more closely related to the way in which a population of the RGCs encodes an external stimulus. Some recent studies have explored the role of recurrence,^39^ using recurrent neural networks (RNNs) to model the shared feature space within the population of neurons. However, the performance of this approach depends critically on the initial location estimate.

To fill in this gap with an explainable model that reveals how the population of neurons work together to encode a larger field of dynamic natural scenes, in this study, we propose that the computations carried out by the retina could be better explained by a convolutional RNN (CRNN) rather than a feedforward CNN. We explore deep CNN and CRNN models with natural scenes consisting of a larger field as input. This approach allows us to determine the shared features of the RGCs and the way they cooperate to encode an external stimulus. The CRNN utilizes many fewer model parameters than the feedforward CNN to directly map out the receptive fields (RFs) of each neuron in the population from dynamic natural visual scenes, producing an outcome that is robust to individual stimulus videos and RFs comparable to those recorded by experiments. Visualization of the results shows that output neurons of the model can learn both the underpinning spatiotemporal RFs of the corresponding RGC and their locations. Furthermore, using a novel pruning strategy for convolution kernels, we find that the CRNN produces a highly effective subset of kernels that capture the performance of the full model. Altogether, these results would inspire researchers to improve the deep-learning strategies for modeling and analyzing dynamic visual scenes.

## Results

### RGC-encoding model with recurrent connections

**Figure 1:**
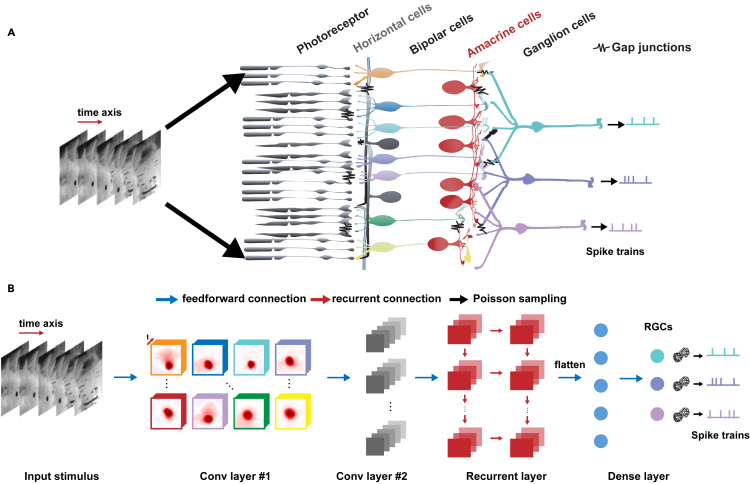
Illustration of the CRNN model architecture (A) Schematic diagram of the retinal circuit. (B) A continuous input stimulus is convolved with the first convolutional layer consisting of several spatiotemporal filters, followed by another convolutional layer that integrates the resulting feature maps. A recurrent layer is incorporated after the last convolutional layer to capture the relationship between the dynamic natural scene stimulus and the retinal response. The activity sequence of the recurrent layer is linearly combined and passed to the final nonlinear activation function for the prediction of the individual RGC responses. Conv layer #1, first convolutional layer; Conv layer #2, second convolutional layer.

In this work, we propose a model consisting of both feedforward convolutional filters and recurrent units. The inputs and target outputs of the model are the natural scene movie stimuli and the responses simultaneously recorded from a population of the RGCs with an electrode array^40^ (see experimental procedures for details of the data). To better study the working principle of the encoding of external input stimuli by the retina, we introduce recurrent connections based on a CNN to get closer to the anatomy of the retina, i.e., the lateral connection, between the RGCs by gap junction or amacrine cells. The CRNN model consists of a four-layer network, including two convolutional layers (model bipolar cells and amacrine cells) that use a rectified linear unit as the activation function. The recurrent connection layer (model lateral connection by gap junction or amacrine cells) is added before the last fully connected layer (model RGCs). The framework of the proposed CRNN model is shown in Figure 1, where one recurrent layer is added to the CNN to capture the temporal dynamics shown in the continuous natural videos and neural responses. The units in the recurrent layer can have a variety of structural forms (e.g., vanilla RNN, LSTM, or GRU, see below for more detailed comparison). Except for special instructions, we use the long-short-term memory (LSTM) units throughout our results. The complexity of the unit structure of the recurrent layer does not have much influence on the performance of the CRNN model.

### RF subunits learned by the encoding models

To quantify the performance of the models, we evaluated the predicting performance of the neural response against various input stimuli, and explored whether the model parameters can conform to one of the critical characteristics of the retinal cell, namely, its RFs. Analysis of the RFs of the hidden layer parameters and the neurons in the last fully connected layer would help us gain a greater understanding of the influence of the recurrent connection layer on model parameter learning.

**Figure 2:**
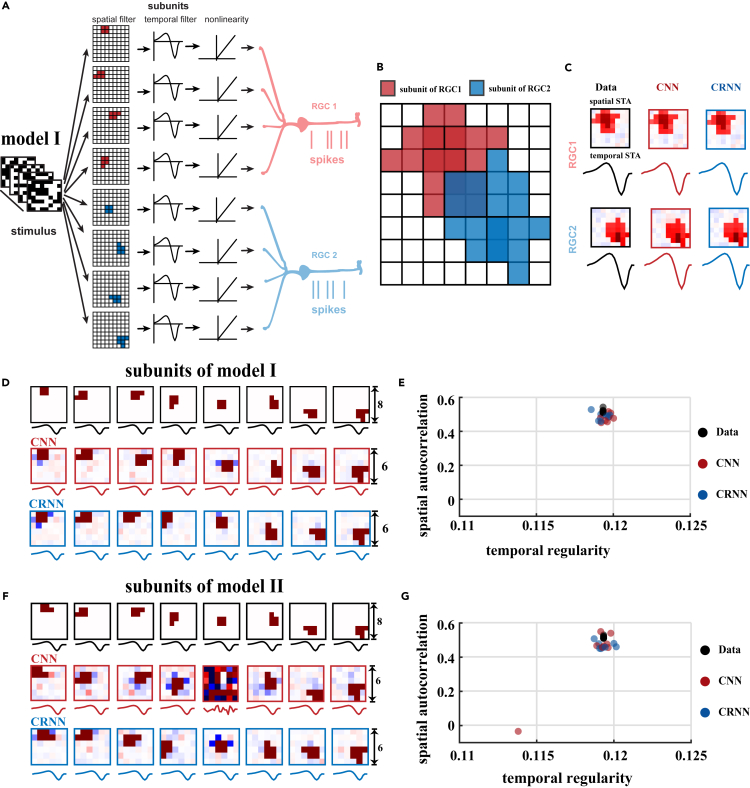
Subunit structures of the two RGC models revealed by the CNN and CRNN models (A) RFs of the two RGCs. (B) Overlaid RF subunits of the two RGCs. (C) Comparison of the measured spatial and temporal STAs of the two RGCs with those predicted by the CNN and CRNN. (D) The RF subunits of model I with the convolutional filters learned in the CNN and CRNN models, with a kernel size of 6. (E) Spatial autocorrelation versus temporal regularity of the model and convolutional filters in the models. (F and G) Similar to (D) and (E), respectively, but for model II, in which the first subunit is changed, followed by altering all eight RF subunits in the models. RF, receptive field; STA, spike-triggered average.

To verify whether the models developed an intermediate computational mechanism similar to a biological retinal circuit by learning the transformation between the input stimulus and the neural response, we generated eight RF subunits and grouped them to create a network model of two RGCs, as shown in Figure 2A, with handcrafted spatial and temporal filters. The RFs of these subunits and the subsequent composition of the RGCs are shown in Figure 2B. To simultaneously encode the response of the two RGC units and their subunits, the first convolutional layer is created with eight spatiotemporal filters, and the dense layer is constructed with two neurons. In addition, 8 × 8 pixel white-noise images are generated as the input of the network. We visualize the RFs of the kernels in the first convolution layer, and the neurons in the dense layer.

To explore the effect of the recurrent connections on the output of the models, we compare the performances of the CRNN and the CNN. To ensure a fair comparison, all the parameters and structure settings of the CNN are kept consistent with the CRNN except for the inclusion of the recurrent layer in the latter. As there is no temporal correlation within the white-noise stimuli, the correlations between the responses of the RGCs and the outputs of both CRNN and CNN reach approximately 0.99 without a significant difference between the models, and both models can learn the spatiotemporal RFs of the two RGCs (Figure 2C), which are computed by the standard techniques of spike-triggered average (STA).^41^ As shown in Figure 2D, the subunits obtained by the CNN and CRNN closely match those given in the model cell. We also altered the size of the kernels in the models and found that we can more effectively map out the RF subunits with relatively large kernels than those with smaller kernels (Figure S1). If the size of the convolution kernel is set relatively small, certain subunits with similar shapes but distributed in different spatial locations can be multiplexed by certain convolution kernels, for example, the subunits shaped like square blocks in the first and fifth subunits.

To further verify the properties of the convolutional kernels learned from the models with different settings, we calculate the spatial autocorrelation as an index of spatial regularity.^42^ We then propose a novel index to describe the temporal regularity (Equation 3 in experimental procedures) of the kernels. Figure 2E shows the distribution of both indices of the RF subunits of model data and for the filters learned in the CNN and CRNN, demonstrating that both models preserved the subunits of the model cell well, indicating that the filters can be effectively learned and that the indices of spatial and temporal regularities can characterize the importance of the RF subunits well.

Our results are robust to the use of $\ell_{1}$ weight regularization, which results in the regularization of the subunits with more compact shapes (Figure S1). Thus, we apply regularization throughout the techniques below. To further examine the robustness of our models, we simulate another network model of the RGCs with different subunit shapes. We manipulated the first subunit (model II) shown in Figure 2F, and found that CRNN is more robust to subunit variations than CNN, in that the CRNN can robustly learn the eight spatiotemporal filters corresponding to the subunits with proper model settings, while the CNN fails to do so to the same degree (Figures 2F and 2G). These results indicate the critical role of recurrent connections in the CRNN to better capture the underlying computational components of the RF subunits of the retinal neural network.

### CRNN enhances the encoding of retinal responses to dynamic natural stimuli

**Figure 3:**
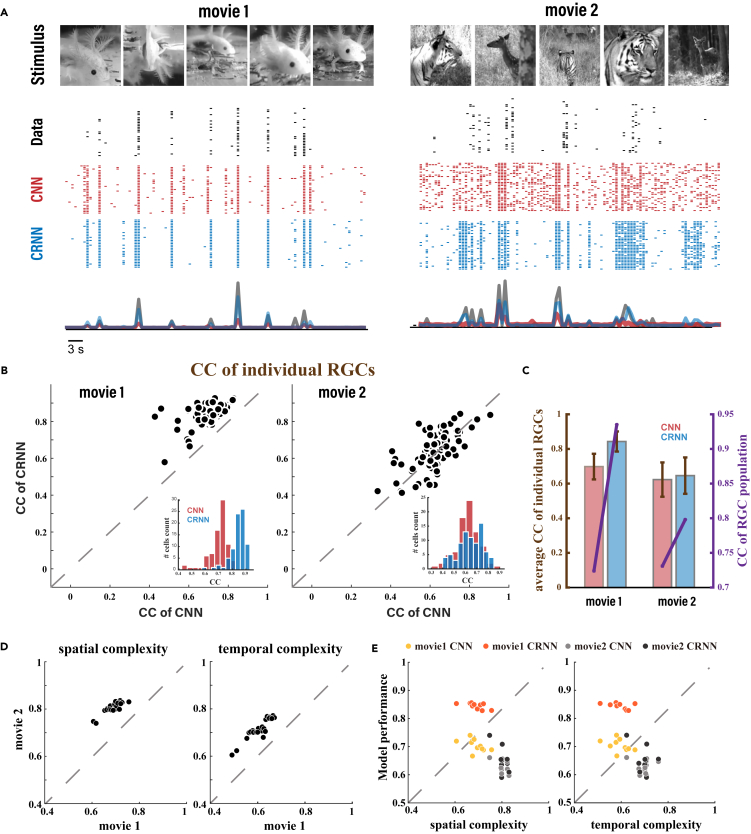
Performance of the models in response to natural movies (A) Spikes and firing rate of the responses of a representative recorded RGC and the predicted CNN and CRNN responses to two natural movies. (B) Scatterplots of the CCs between the electrophysiological data of all RGCs and the responses of both CNN and CRNN models to the two movies. (C) Average CC in response to both movies between the RGCs and the models (left y axis), and CC between the population RGC response and the average of the output of models (purple line belongs to the right y axis). (D) Different levels of complexity between the two movie stimuli. Each dot represents a slice patch used for computing the correlation. (E) Relationship between the complexity of the movies and the performance of the models. The points in (B), (D), and (E) refer to different cells.

To verify the performance of the CRNN model regarding the electrophysiological data and evaluate whether the models can learn the adaptability of the retina to dynamic visual scenes, we further built models for the prediction of the response of a population of RGCs to natural movies. Two natural movies approximately 60 s long were used to train the models. The first movie (movie 1) was relatively simple, consisting merely of scenes of salamanders swimming in a tank. In contrast, the second (movie 2) was more complex, showing a tiger hunting its prey, in a backdrop of grass and trees, and with fast transitions between scenes. Example frames of the two natural movies and the corresponding RGC responses in terms of individual trials of spike trains as well as trial-averaged firing rate are shown in Figure 3A, together with the model output in the format of firing rate, from which one can sample individual spikes using the Poisson process (Table 1).^38^

After ***h***_*t*_ network training, we evaluated the correlation coefficient (CC) between the response of each RGC and the output of the neurons in the dense layer, as well as between the average firing rate of the output neurons in the models and the average response of the RGCs. The performance of the models with respect to the RGC population is shown in Figures 3B and 3C, where the CRNN markedly outperforms the CNN on both movies (average CC of RGCs 0.857 versus 0.698 on movie 1, 0.718 versus 0.623 on movie 2), independent of the size of the training data (Figure S2). This improvement is also true when training the models with individual trials of spike trains (Figure S3). Particularly, for movie 1, the CRNN performs notably better than CNN, which may be because movie 1 is visually less complex than movie 2. Subsequently, to evaluate the complexity of dynamic natural scenes quantitatively, we characterized the spatial and temporal complexities of the scenes by calculating the structural similarity index (SSIM) between patches of the frames of movies, and compared them between the two movies in Figure 3D, which indicates that movie 2 was more complex than movie 1. We then examined the relationship between the complexity of the dynamic visual scenes (see Figure S4 and experimental procedures) and the performance of the models in terms of the CCs between the individual RGCs and the model outputs. As shown in Figure 3E, the performance of the models was lower for the more complex movie 2 than for movie 1, which suggests that the complexity of the visual scenes is indeed a major driving force for modeling prediction.

### CRNN recovers the neuronal RF using dynamic natural scenes

**Figure 4:**
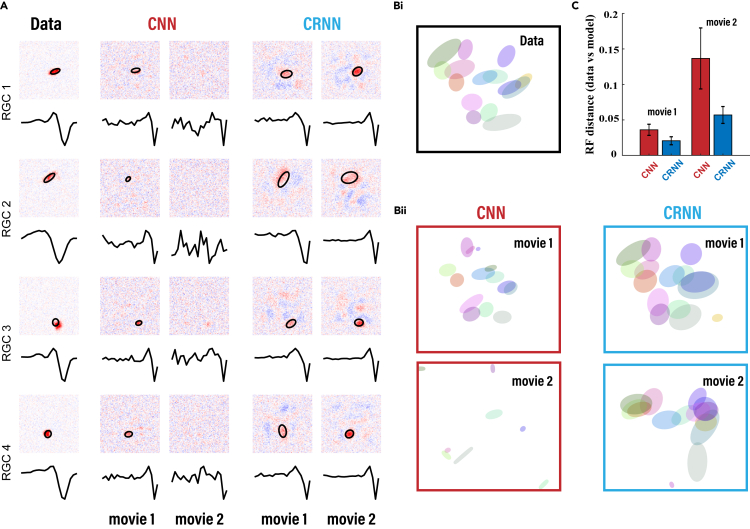
The CRNN reproduces the spatial RFs and temporal filters of RGCs (A) The spatial RFs and temporal filters of four representative RGCs and the corresponding neurons learned by the CNN and CRNN. The ellipses indicate the RFs fitted by a 2D Gaussian function. (B) The RFs of 15 RGCs randomly selected from a population of 80 cells from the dataset (i) and those generated by the models (ii) computed from white-noise stimuli. (C) RF distance between the data and the models for all RGCs.

In addition to evaluating the ability of the model to predict responses, we further show whether the structural components of the models can capture the intermediate computational mechanism of the retinal encoding circuit for dynamic scenes. Figure 4 shows the RFs of the RGCs and those that the CNN and CRNN learned when trained on movie 1 and movie 2. Experimentally, the RFs were computed with the STA obtained from white-noise stimuli to obtain a 3D spatiotemporal RF filter. Thereafter, we applied singular-value decomposition (SVD) to the 3D filters to obtain a temporal filter and spatial filter (Figure 4A). Two-dimensional Gaussian functions were fitted to the components of the spatial RFs obtained from both data and models to determine their center, size, and shape. Figures 4A and 4B show the fitted 2D Gaussian function of each RGC and model neuron as ellipses.

To better quantify the similarity of the RFs between the RGC data and the models obtained for each cell, we calculated the cosine distance between the 2D Gaussian distribution of the RFs of the model neurons and those of the recorded RGCs (Figure 4C). While calculating the RF distance, we considered only the neurons that are able to learn an RF. For example, the neurons indicated in the third column of Figure 4A, which do not learn an effective spatial RF, are not included in the statistical analysis. The model with the best performance (the CRNN model trained on movie 1) best reproduced the RFs of a large number of the RGCs with a notably small distance between the RFs of the data and the model, while the CNN model trained on movie 2 was unable to learn either the spatial or the temporal filters. Moreover, as the temporal correlations in movies of natural scenes are much higher than those found in white-noise stimuli, the temporal filters obtained by the models trained on these movies adapted better than the filters calculated by white-noise stimuli. The models that learned the temporal filters usually produced filters whose first peak had a low temporal latency, while the peaks of the temporal filters obtained using white-noise stimuli had much longer latency, which is a peculiar feature of the temporal adaptation induced in the retina by stimulus images with different statistics.^43^ These results indicate that the CRNN, and not the CNN, can model the rich computational structures of the retinal neural circuit while learning the complex dynamic visual scenes.

### Efficient learning of the CRNN model

In the previous sections, we have described how the introduction of the recurrent layer can improve the model performance in predicting the retinal response to dynamic sequence stimuli and the robustness of learning to infer the subunits. In addition to the response prediction performance, quantification of the effectiveness of CRNN on the retinal electrophysiological data is a key issue in evaluating a neural coding model. We compare the CNN and CRNN from two aspects: inferring the subunits of the retinal circuit and learning to predict the responses of large-scale population ganglion cells. They are used to evaluate whether the introduction of the recurrent layer can improve the effectiveness of the retinal coding model.

**Figure 5:**
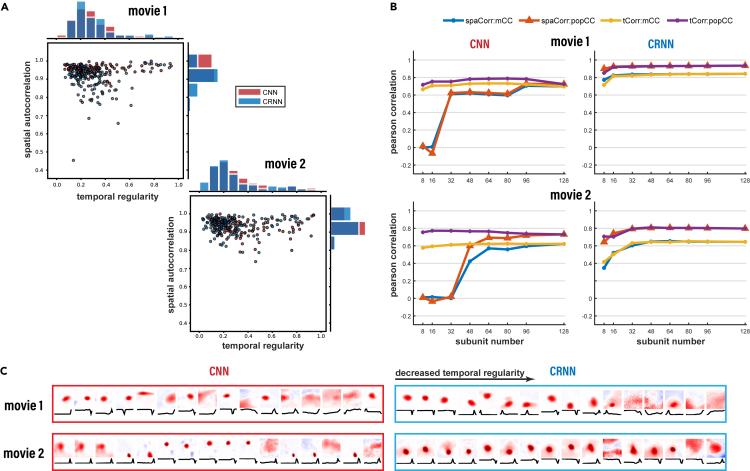
Model construction using effective components with highly temporal regularity or spatial autocorrelation (A) Distribution of the spatial autocorrelation and temporal regularity indices of all filters learned from both movies by the CNN and CRNN. (B) Performance (CC) on both movies with reduced models incorporating rank-selected filters. The average individual CCs (mCC) and the population response CC (popCC) between the data and the pruned models are shown. (C) Convolutional filters of the CNN and CRNN models sorted by decreasing the temporal regularity. spaCorr, spatial autocorrelation; tCorr, temporal regularity.

First, we evaluated the kernel parameters of the first convolutional layer trained on the movies according to the subunit importance indices, the spatial autocorrelation, and the temporal regularity (shown in Figure 5A). The spatial autocorrelation can measure whether the spatial filter of the convolutional kernel is relatively concentrated in a certain area, while the temporal regularity can measure the adaption regularity of the temporal filter. When we construct reduced/pruned models using fewer subunits selected according to the value of either index, the performance is found to be better preserved in models pruned based on the temporal regularity index. The pruning results with different numbers of convolutional filters quantified by both the spatial and the temporal indices are shown in Figure 5B. The performance of the reduced models can be maintained at a good level regardless of the temporal regularity indices of the remaining subunits. In contrast, when using the spatial autocorrelation, the performance of the reduced CNN model significantly drops when the number of convolution kernels is less than 32. Moreover, it is interesting to note that by using temporal filter regularity as a quantified index, the reduced CNN model can still achieve high performance on both movies. For the CRNN, the convolution kernels learned in the model are better than those in the CNN model; thus, the prediction performance can be maintained at a better level when unimportant convolution kernels are removed, especially for the model with the best performance in movie 1. A few examples of selected filters sorted in terms of decreasing temporal regularity are illustrated in Figure 5C, indicating that the filters are more organized in movie 1 than in movie 2. Altogether, these results signify that the temporal regularity is prioritized in learning dynamic visual scenes, and the CRNN enables us to implement efficient learning with a superb representation of the retina, even with a much smaller set of learned parameters.

**Figure 6:**
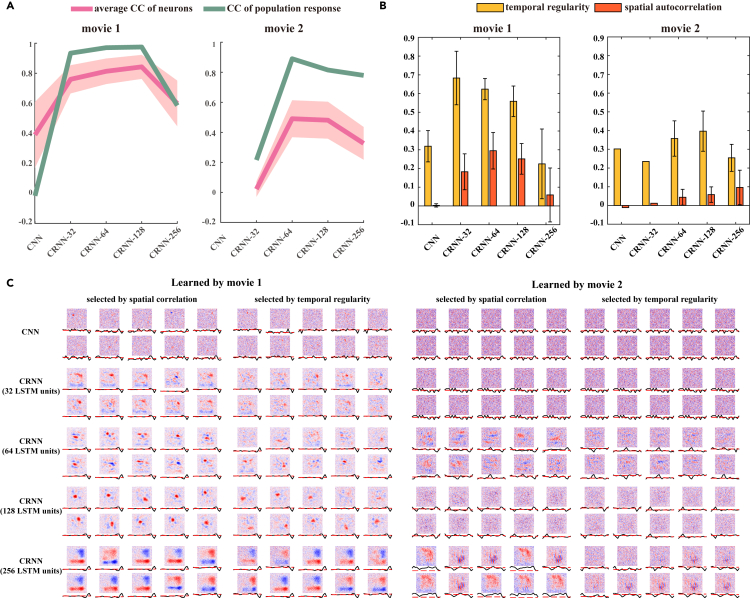
Model behavior of a large population of RGCs (A) Performance of CNNs and CRNNs with different numbers of recurrent units. Red and green indicate the average CC of individual cells and the CC of the population response, respectively. (B) Distribution of the temporal regularity and spatial autocorrelation of the RF filters from all neurons learned from the different models. The error bars represent SD. (C) Examples of the RFs learned from the different models. For each model, 10 spatial and 10 temporal filters were selected and ranked by the spatial autocorrelation and temporal regularity, respectively, for each movie.

Thus far, we have used a population of 80 RGCs from a single recording to serve as our electrophysiological dataset. To further explore the effect of the amount of data size on model learning, we obtained a second dataset involving 14 recordings from 1,218 RGCs for movie 1 and 500 RGCs for movie 2, and used this dataset to again train the models. In addition, we evaluated the influence of the number of hidden units in the recurrent layers on the performance of the models. Table S1 shows the number of parameters used in the models constructed for this set of experiments: the existing CNN model described above and CRNN models constructed with 32, 64, 128, and 256 recurrent units. Following the training of these models using the second dataset mentioned above, the CRNN models were found to outperform the CNN models in both movies (Figure 6A), similar to the results reported in the above subsections. However, we found that more recurrent units are not always better; eventually, a large number of recurrent units result in a deteriorated model performance. For movie 1, the CRNN model achieved good performance with 32 recurrent units. As the number of units increased to 64 and then 128, the performance of the CRNN models slowly increased. For movie 2, the CRNN model did not perform well with 32 recurrent units and achieved the best performance with 64 units, implying that the optimal CRNN model for movie 2 requires 64 recurrent units, while the performance for movie 1 is relatively equitable with 32–128 units.

To examine the RFs learned by these models, we first computed them using white-noise images as described earlier, followed by calculating the spatial autocorrelation and temporal regularity of the RFs of the output neurons. The average values of these indices are shown in Figure 6B. Some examples of the spatiotemporal RFs learned by the models with the highest spatial autocorrelation (left) and temporal regularity indices are shown in Figure 6C. The CRNN models with the best performance on movie 1 (CRNN-128) and movie 2 (CRNN-64) exhibit more regularized RFs (spatial centralized, regular oscillatory temporal wave) than other models. For the CRNN with 256 recurrent nits, the centralized area of the spatial filters was much larger than that of other models, while the spatial filter had less diversity, or in other words, was more uniform. This observation suggests that using more recurrent units can yield output neurons with similar RFs, which can be combined with several small RFs in the same region. In addition, when training with the complex scenes of movie 2, some spatial RFs of the CRNN exhibited complex tuning beyond spatially localized center-surround tuning, which could be due to the overly dense representation in the collated population of the RGCs used for training the model. Taken together, these results suggest that a CRNN with more RGC samples could achieve a nearly 100% perfect performance, while a feedforward CNN would be unable to learn the response of a large group of RGCs. Moreover, the CRNN could be capable of demonstrating robust performance with a small number of recurrent units.

### Different structures of the recurrent layer

**Figure 7:**
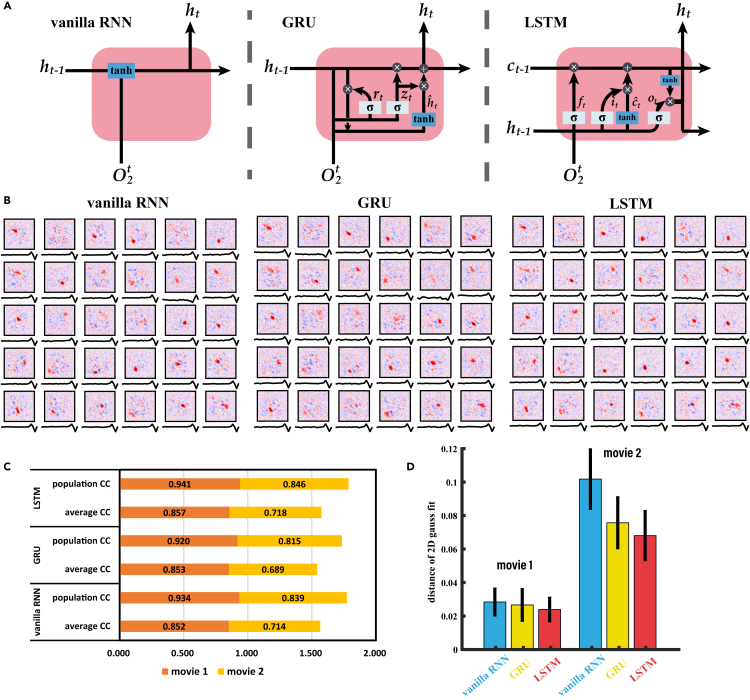
The CRNN models with different recurrent layers (A) Structures of different kinds of recurrent units. (B) Spatial RFs and temporal filters of neurons learned by the CRNN with vanilla RNN, GRU, and LSTM. (C) Performance of the CRNN models based on different recurrent units to predict the neural response to two natural video stimuli (movie 1 and movie 2). Population CC, the correlation coefficient between population RGCs and the average output of the models; average CC, the correlation coefficient between the RGCs and the models' outputs. (D) RF distance of each cell between the data and the model outputs.

To show that our results are not dependent on one specific type of recurrent structure, we tested and compared three types of recurrence: vanilla RNN, gated recurrent unit (GRU),^44^ and LSTM.^45^ Their structures are shown in Figure 7A. The predicting performances of these three structures for natural movies are similar (Figure 7C). In addition, we examined whether the models could obtain the spatiotemporal RF of the RGCs, and some example results are shown in Figure 7B. By calculating the cosine distance between the 2D Gaussian distribution of the RFs of the models and those of the recorded RGCs, we found that the models with LSTM units could obtain RFs with higher similarity with the RGC data (Figure 7D). Overall, these results demonstrate that CRNN models with different kinds of recurrent units can achieve comparable performance and outperform the CNN model. In other words, the difference in performance between CNN and CRNN is not due to the complexity of the LSTM/GRU units, but the recurrent layer is essential. However, considering the maintenance of the model's ability to learn long-term input stimuli, the vanilla RNN models are prone to gradient disappearance/explosion when receiving long-term stimuli, and the performance of the model based on LSTM is better than that based on GRU, as well. Therefore, the results of the CRNN models obtained in the above section were constructed with the LSTM. These results indicate that the recurrent layer as a general form, rather than specific models of recurrent units, plays a functional role in explaining neural responses to dynamic visual scenes.

## Discussion

To unravel how the retina computes dynamic natural visual scenes, we have investigated the role of recurrent connection in encoding complex dynamic visual scenes in the retina in this study. Using both simulated and experimental data, we observed that the CRNN was more effective than the CNN in predicting the response to the movies, as well as at generating the effective and comparable RFs shown in experiments. This observation, independent of the specific choice of recurrent units modeled, is a general feature that emerged from the neural response to dynamic visual scenes.

### The role of the recurrent connection

The CRNN models capture several important properties of the biological retinal circuits, while the CNN cannot. First, the convolutional filters in the first hidden layer of the CRNN are more consistent with the model subunits in terms of the shapes of the RFs, regardless of kernel size changes. Second, the CRNN better predicts the response of the animal retina to natural scene movies. Last, the CRNN provides an estimate of the location and shape of the RF of each RGC.

Inspired by experimental observation in neuroscience,^46^ typical neural networks have a hierarchical architecture with several layers. Some of these layers include a block of convolutional filters, and consequently, each filter serves as a feature detector to extract an important property of the input images.^47^ Thus, training with a large set of images allows the convolutional filters to play functional roles as neurons in the retina and the elements of other visual systems to encode the complex statistical properties of natural images. The filter shapes are sparse and localized, like the RF of the visual neurons. Therefore, it would be reasonable to use similar neural network approaches to investigate the central question of neuronal encoding in neuroscience.^8^

In visual coding, the ventral stream of visual processing in the brain starts in the retina and passes to the lateral geniculate nucleus via the optic nerve, and finally the layered visual cortex, to reach the inferior temporal gyrus. This visual pathway has been suggested as the “what pathway,” used to recognize and identify visual objects.^48^^,^^49^ A deep neural network was used to model and predict with reasonable accuracy the activity of the neurons in the inferior temporal cortex in monkeys.^8^^,^^50^, ^51^, ^52^ Therefore, the biological underpinnings of the ventral stream of visual processing in the brain can be related to the structure components used in deep neural networks. However, interpretation of this relationship is not straightforward, since the pathway from the retina to the inferior temporal cortex is complicated,^8^ although in the retina the neuronal organization is relatively simple.^53^

Previously, a few studies have taken this approach by applying different kinds of CNNs to model earlier elements of the visual systems on the brain, such as the retina,^15^^,^^39^ V1,^54^, ^55^, ^56^, ^57^, ^58^ and V2.^59^ Similar to the current study, most of these studies sought to demonstrate that a better performance in terms of neural response could be achieved by using either a feedforward CNN or an RNN, or both. The results presented in this study provide a promising direction in which to reinvestigate the functional role of feedforward and recurrent approaches for different types of visual scenes. The recurrent layer plays an important role in modeling neuronal nonlinearity, which is a unique feature of neural computation.^60^ By incorporating recurrent connections, many models have shown advantages in recognizing static images.^22^^,^^25^^,^^61^, ^62^, ^63^, ^64^ An unrolled recurrent network is equivalent to a deeper or wider network that saves on neurons by repeating data transformation several times,^24^^,^^65^^,^^66^ but it improves the flexibility trading of speed and accuracy in biological vision.^67^ Our results, together with those from other recent studies, provide new insights into the underlying mechanisms of neuronal encoding for dynamic visual scenes, as well as the design of better models for analyzing dynamic visual scenes.

Another potential approach is to model recurrence with an additional layer of neurons to mimic the role of inhibitory amacrine cells of the retina. One simple way is to add inhibition using local normalization, such that each inhibition cell suppresses a local group of cells, similar to the retinal biologic mechanism. However, how to add grouping constraints to make lateral suppression work is still an open problem. As early as in the AlexNet^68^ on the image classification task, the authors introduced local response normalization to make neurons in the same local region inhibit one another. However, the divisive normalization did not bring too much gain to the network performance. Hence, this idea is not widely used in deep neural networks so far. Given the rich neuroscience knowledge from the retina and other visual pathways, there may be other ways to add inhibitory neurons to artificial neural networks. It is worthy of more detailed investigation and future work.

### Model parameter pruning using temporal filters

To evaluate whether the elements of the models play the roles of intermediate computational mechanisms similar to those used in biological retinal circuits, several subunit importance evaluation indices have been introduced. In one study,^17^ the authors reduced the number of hidden neurons or stimulus attribution of the trained models based on an importance index and finally tested the effectiveness of CNN subnetwork models through their response-based performance in experimental protocols, including omitted stimulus response, motion reversal, etc. This method is used to quantify the importance of the model units according to their contribution to the final neuronal firing rate and exploit stimulus invariance to reduce computational dimensionality. However, this is infeasible when the natural dynamic stimulus is not spatially invariant, and the prediction result is a population response rather than a single neuron response; furthermore, it is difficult to measure the contribution of each subunit to the individual RGCs.

In other studies, the effectiveness of model subunits has been quantified by the spatial autocorrelation of the convolution kernels,^16^^,^^42^ and was determined by the Moran index. However, this method can be verified only on the white-noise stimuli, since they are not spatiotemporally correlated. Hence, the spatial RF of the convolution kernel is relatively concentrated in a small area, representing only the center effect of the RF without the surrounding inhibition effect.^41^ Such a simple RF allows the selected subunit to achieve desirable results. However, in dynamic natural scenes, each pixel has a comparatively high correlation with spatially adjacent areas, leading to a large spatial autocorrelation for the convolution kernel, with no possibility of reduction of the correlation as represented in the Moran index. Consequently, we took advantage of the biphasic response via the ON and OFF polarity in the temporal filter of the RF produced by temporal adaption^43^^,^^69^ and evaluated it according to a relatively regular oscillatory wave with some peak sensitivity and period of adaption. Temporal adaption is ubiquitous not only for the neural computation of sensory input,^43^ but also for controlling and adjusting the dynamic range of single cells and neural populations in investigations of general neural dynamics.^70^^,^^71^ Without limitation of the properties of visual stimulus, e.g., spatial invariation, our importance index, an important feature for modeling of the retinal encoding, used to evaluate the temporal filter enables us to incorporate neuronal adaption in response to complex and dynamic natural visual scenes.^72^^,^^73^ Thus, our filter pruning approach could help reduce the effective number of parameters in other deep-learning models while processing the dynamic visual scenes, expanding their performance beyond that for static natural images.

### Application to other systems

Here we use the retina as a model system to explain the role of recurrence in the network modeling the relationship between neural response and dynamic visual scenes. It is well known that the retina is one of the best-understood examples in neuroscience for visual computing.^1^ The methodologies for the retina generally work well for other visual pathways, from the lateral geniculate nucleus and primary visual cortex to the inferior temporal cortex, as well as neural coding in other parts of the brain.^8^^,^^38^ Recent studies also emphasize the role of recurrence in visual computing.^20^^,^^63^^,^^64^ Our work aligns with this line of showing the unique feature of recurrence in neural network models: the recurrent connection plays a role similar to maintaining the *memory* of lasting external stimulation and ongoing neural dynamics. In other words, the recurrent layer stores the previously input computation information in the hidden node state, and then a new prediction judgment can be made by combining the previously stored information when a new input is introduced. This functional role is generally shown in various brain areas.^7^^,^^8^

The topic of our work focused on dynamic visual scenes, e.g., continuous videos. Video analysis is of great interest to data science researchers, not only for neuroscience, but also for other domains of applied vision, including machine vision, neuromorphic computing, and brain-machine interface, where a large chunk of data in the format of videos is analyzed.^2^ Analysis of static natural images is relatively easy,^18^ while videos span multiple scales in space and time, which raises tremendous difficulties for analyzing the contexts themselves,^74^ as well as for characterizing the underlying neural dynamics.^72^ We show that movies with different levels of complexity show different behaviors in models. Such difference calls for a further investigation of the level of scene complexity and how it affects neural dynamics and the network modeling approach.

Visual neuroscience is an immensely popular topic in machine learning, such that numerous methodologies developed have a broad application to and inspiration for other topics.^7^^,^^46^ From the perspective of deep learning, the introduction of recurrent connections affects the parameter adjustment of the model during backpropagation, subsequently affecting the learning results. When modeling the neural response of simulated data, the convolution kernels of the CRNN are more consistent with neural subunits. When the model is trained with videos, the spatial autocorrelation and temporal regularity of the spatiotemporal filters in the CRNN model are stronger, suggesting that the lateral connections routed by inhibitory cells or gap junctions play a functional role. In particular, it is very beneficial to use temporal regularity to reduce the model parameters to a subset while maintaining the model performance. It implies that the effect of the temporal domain in videos is more prominent than that of the spatial domain.^43^^,^^74^ These implications based on a neuroscience-inspired approach could provide inspiration for algorithm designs of artificial intelligence.^46^

### Limitations

In this work, the CRNN model mainly simulates the structure of the retina, with a three-layer feedforward network with some interneurons and gap junctions. Thus the proposed model was studied to simulate the encoding process of the retina with a relatively simple setting. However, in addition to the interneurons such as amacrine cells and gap junctions, there are other interactions between cells in the retinal circuit, for instance, feedback from RGCs to the inner retina.^75^ The current architecture of our proposed models is based on the simple assumption of the retinal circuit. These retinal components that we have not simulated may play an indispensable role in the process of encoding the external environment by the retina. Future work is needed to include these feedback factors, which can improve our modeling approach beyond the retina to other higher visual pathways.

When learning to predict the response of the same 80 ganglion cells to the two natural scene videos, CRNN can train an effective model on each stimulus. However, it is still unable to transfer the models between videos, e.g., a model trained on movie 1 cannot predict well the response of RGCs to movie 2. Thus, future work is needed to introduce some strategies to make the model show good generalization performance on different visual stimuli. Furthermore, dealing with the higher complexity of movies may need additional mechanisms. One possible mechanism is attention or feedback, which has engendered significant efforts in modeling visual computing.

The added recurrent layer could have an attention mechanism whereby the recurrent unit will pay attention to different features of the input. Except for the recurrent-based attention network, recently the transformer,^76^ based solely on attention mechanisms, has outperformed many convolutional recurrent networks on processing sequential tasks, e.g., natural language processing. Ideally, in the future, we hope to build models utilizing these deep-learning architectures for neural encoding and decoding of the visual pathway rather than the retina. Another direction is to use graph neural networks processing non-Euclidean data.^77^ The current models of retinal coding usually receive frame-based input, which belongs to the distribution of the Euclidean domain. The traditional convolutional layer can extract features well on regular Euclidean data. In the future, we will explore how to build a model to predict the retinal response against stimuli that belong to different data distributions in the non-Euclidean domain. These strategies could reinforce our consideration of the role of recurrence in visual computing, either data raised from neuroscience or applications of neuromorphic computing, brain-machine interface, and video analysis.

## Experimental procedures

### Resource availability

#### Lead contact

The lead contact for this study is Jian K. Liu: j.liu9@leeds.ac.uk.

#### Materials availability

This study did not generate new unique materials.

#### Data and code availability

The data are available at https://doi.org/10.5061/dryad.4ch10. The code is available at https://github.com/Zyj061/retina-crnn_model.

### The RGC encoding models

To untangle the underpinnings of the retinal system and gain a clearer understanding of the stimulus/response relationship for dynamic scenes, we propose two deep-learning models to describe RGC encoding: one based on a CNN and another based on a CRNN.

#### CNN

The structure of the proposed CNN encoding model is the same as that of the models used in the references.^14^^,^^16^ In the CNN model, we establish two convolutional layers to extract the spatiotemporal information of the input stimulus. The outputs of these convolutional layers are obtained as follows:

(Equation 1) $$O_{l}=g\left(\varphi \left(W_{l}\text{∗}O_{l-1}+b_{l}\right)\right)\text{,}$$

where $W_{l}$ and $b_{l}$ are the convolutional weights and biases of layer l, respectively. ∗ denotes the convolution operation, and g(⋅) is the activation function, which is set as the ReLU function g(x)=max(0,x) in this work. φ(⋅) denotes all the operations that follow the convolution, e.g., batch normalization and the addition of Gaussian noise. After the second convolutional layer, we flatten the output $O_{2}$ into a one-dimensional vector ${\tilde{O}}_{2}$, and pass it through a dense layer with n output neurons corresponding to the population RGCs. The outputs of the dense layer denote the firing rates of the RGCs, which are obtained as follows:

(Equation 2) $$\hat{y}=\varphi \left(W_{d}\cdot {\tilde{O}}_{2}+b_{d}\right)\text{,}$$

where φ(x) is the parametric softplus function φ(x)=α⋅log(1+exp(βx)), and $W_{d}$ and $b_{d}$ are the connected weights and biases, respectively, of the dense layer. The α and β are trainable parameters. Taking the actual firing rate y of the RGCs as the fitting target, the models are optimized to jointly minimize the Poisson loss function and regularization as follows:

(Equation 3) $$L\left(y,\hat{y}\right)=\frac{1}{N}\left(\hat{y}-y\text{log}\hat{y}\right)+{\left||W_{d}|\right|}_{2}+{\left||\hat{y}|\right|}_{1}\text{,}$$

where N is the batch size of the samples used at each iteration. ${\left||\cdots |\right|}_{1}$ and ${\left||\cdots |\right|}_{2}$ represent $\ell_{1}$ and $\ell_{2}$ norm regularization, respectively. To avoid overfitting and ensure that the neurons are sparsely firing, we apply $\ell_{2}$ norm regularization to the weights and $\ell_{1}$ norm regularization to the neuron activation. In each layer of the network, we add the $\ell_{2}$ norm to regularize the weights of the layer. The output of each layer is normalized using batch normalization prior to the nonlinear activation function. In the last fully connected layer, the softplus activation function is used with trainable parameters α and β. The weight of the first convolutional layer is regularized by the $\ell_{1}$ norm to let the RFs of the convolutional kernels have more compact shapes. The nonnegative loss and Adam optimization strategies are used for multivariate regression training.

#### CRNN

In the CRNN model, we add an additional recurrent layer between the second convolutional layer and the final dense layer of the previous CNN model. We have examined the effects of different kinds of units in the recurrent layer on predicting the retinal response to natural movie stimuli, including vanilla RNN, GRU, and LSTM. We use 32 recurrent units as the components of the special recurrent layer (the number of recurrent units can be adjusted when the number of RGCs increases), which has been shown to be powerful and efficient in modeling sequence dependencies. We take the output of the second convolutional layer $O_{2}=\left\{O_{2}^{1},\ldots ,O_{2}^{t}\right\}$ as the input sequence to the recurrent layer, where each feature map $O_{2}^{t}$ is the input at each time step. In the following, we introduce the details of vanilla RNN, GRU, and LSTM.

##### Vanilla RNN

In the vanilla RNN unit, output state vector $h_{t}$ is obtained by passing through the multiplication of the output of the second convolutional layer $O_{2}^{t}$ and the previous state $h_{t-1}$ to the **Tanh** activation function:

(Equation 4) $$h_{t}=\text{tanh}\left(W\cdot O_{2}^{t}+U\cdot h_{t-1}+b\right)\text{,}$$

where W, U, and b are the feedforward weight matrix, recurrent weight matrix, and bias vector, respectively, which need to be learned during training. W, U, and b in the following formulas also have the same meaning.

##### GRU

Compared with the vanilla RNN, update gate $z_{t}$ and reset gate $r_{t}$ are introduced into the GRU unit to avoid gradient vanishing/exploding with long-term stimuli. The hidden state $h_{t}$ of the GRU unit is obtained by:

(Equation 5) $$z_{t}=\sigma \left(W_{z}\cdot O_{2}^{t}+U_{z}\cdot h_{t-1}+b_{z}\right)\text{,}$$

$$r_{t}=\sigma \left(W_{r}\cdot O_{2}^{t}+U_{r}\cdot h_{t-1}+b_{r}\right)\text{,}$$

$${\hat{h}}_{t}=\text{tanh}\left(W_{h}\cdot O_{2}^{t}+U_{h}\left(r_{t}\circ h_{t-1}\right)+b_{\hat{h}}\right)\text{,}$$

$$h_{t}=\left(1-z_{t}\right)\circ h_{t-1}+{\hat{h}}_{t}\circ z_{t}\text{,}$$

where ⋅ refers to dot production, and ∘ denotes element-wise multiplication.

##### LSTM

Each LSTM unit consists of an input gate $i_{t}$, a forget gate $f_{t}$, and an output gate $o_{t}$, while one hidden unit means maintaining one time-step memory at t. The states of these gates and cells are as follows:

(Equation 6) $$f_{t}=\sigma \left(W_{f}\cdot O_{2}^{t}+U_{f}\cdot h_{t-1}+b_{f}\right)\text{,}$$

$$i_{t}=\sigma \left(W_{i}\cdot O_{2}^{t}+U_{i}\cdot h_{t-1}+b_{i}\right)\text{,}$$

$$o_{t}=\sigma \left(W_{o}\cdot O_{2}^{t}+U_{o}\cdot h_{t-1}+b_{o}\right)\text{,}$$

$${\hat{c}}_{t}=\text{tanh}\left(W_{c}\cdot O_{2}^{t}+U_{c}\cdot h_{t-1}+b_{c}\right)\text{,}$$

$$c_{t}=f_{t}\circ c_{t-1}+i_{t}\circ {\hat{c}}_{t}\text{,}$$

$$h_{t}=o_{t}\circ \text{tanh}\left(c_{t}\right)\text{,}$$

where ${\hat{c}}_{t}$, $c_{t}$, and $h_{t}$ represent the cell input activation vector, cell state vector, and output vector, respectively, and ∘ denotes element-wise multiplication.

Each recurrent unit generates a sequence output $h=\left\{h_{1},\ldots ,h_{t}\right\}$ *t* = 64, and by stacking $n_{l}=32$ recurrent units in one layer, the output of the entire recurrent layer is $H=h^{1},\ldots ,h^{n_{l}}$. Similar to the outputs of the convolutional layer, we also flatten the recurrent layer output H into a one-dimensional vector $\tilde{H}$, and pass it through a dense layer with n output neurons. The final output of the CRNN model $\hat{y}$ is the following:

(Equation 7) $$\hat{y}=\varphi \left(W_{d}\cdot \overset{˜}{H}+b_{d}\right)\text{,}$$

where φ(⋅) is identical to the parametric softplus function used in Equation 2. Similar to the training process of the CNN model, the CRNN model is optimized by minimizing the Poisson loss, with $\ell_{1}$ regularization on neuron activity $\hat{y}$ and $\ell_{2}$ regularization on the connected weight $W_{d}$.

#### Model implementation

All of the models were implemented with Keras using TensorFlow as the back end and trained on NVIDIA K80 GPUs. The training epoch was set to 1,000, but the training would terminate early if the loss converged. To model biophysical RGC responses using an entire frame as input, we used a filter size of 25 × 25 in the first convolutional layer and a filter size of 11 × 11 in the second layer. For the CRNN models referred to in the results, which were trained on natural movies, we used LSTM units in the recurrent layer with $\ell_{2}$ norm regularization in the kernels. In addition to modeling larger amounts of RGC data with CRNN models constructed with 32, 64, 128, and 256 LSTM units, we used 32 units in all the other CRNN models. Except for the recurrent layer, all the other units and hyperparameters were the same for the CNN and the CRNN. The settings of the CNNs and CRNNs used for learning the relationships between the dynamic responses of the population RGCs and the natural movies are shown in Table 1.

**Table 1:** Models’ parameter settings

Name	Description	Size/value
Inputs	spatiotemporal stimulus	90 × 90 × 20
conv1 num	number of kernels in the first convolutional layer	128
$\ell_{1}^{1}$	weight regularization in the first convolutional layer	5 × 10^−4^
conv2 num	number of kernels in the second convolutional layer	64
$\ell_{2}$	weight regularization	10^−3^
$\ell_{1}$	activity regularization on the dense layer	10^−3^
Outputs	dynamic responses of the population RGCs	80

#### Model pruning

To examine whether the deep-learning-based mode just learns the relationship without explainable hidden units, we developed a novel pruning strategy to evaluate the importance of the parameters of the convolution kernels, and to evaluate whether the models act as intermediate computational mechanisms similar to those used in biological retinal circuits. According to previous analyses of temporal filters of neural circuits, the RF of an effective temporal filter is a relatively regular oscillatory wave with a certain peak sensitivity and an adaptation period, such as that for ON or OFF bipolar cells.^69^ Therefore, we propose a novel subunit importance index to quantify the wave regularity of a temporal filter. The calculation formula is as follows:

(Equation 8) $$I_{temporal}=\frac{1}{T}\sum_{i=1}^{T}\left|\left\|w_{i}|-\text{max}|w\right\|\right|-\frac{\varepsilon }{w-{\bar{w}}_{2}+\varepsilon }\text{,}$$

where w is the weight of the temporal filter obtained by SVD of the first convolutional kernel, T is the length of the temporal filter, and $w_{i}$ is the element of the temporal filter at position i. The first term of the formula determines whether there are regular wave peaks in the temporal filter. To eliminate the influence of the corresponding ON and OFF subunits, the first term is calculated using the absolute value of the given parameter of the kernel. In the second term, the Euclidean distance between each temporal filter parameter and its average value is used as the denominator to improve the diversity of the temporal filter. ε is a small value, which is set to 5 × 10^−4^.

### RGC experimental data

To verify the performance and effectiveness of our models with biological data, we used public datasets recorded from the ganglion cells of isolated salamander retinas using multi-electrode arrays with natural movies as the input stimuli.^40^ Briefly, each frame of the movies covered an area of 2,700 × 2,700 μm^2^ on the retinas with a spatial resolution of 360 × 360 pixels. The multi-electrode arrays were used to record the responses of 80 RGCs to 31 and 33 trials of the presentation of movie 1 (simple scenes of swimming salamander) and movie 2 (complex scenes of animals), respectively (described earlier under “CRNN enhances the encoding of retinal responses to dynamic natural stimuli”). For model training, the target output was created by averaging the response from each cell over all trials and binning with a bin width of 33 ms. To have the model learn the spatial and temporal filters, at each of the time bins created for the target model output, the corresponding frame of the movie downsampled to 90 × 90 pixels, along with the frames of the preceding 20 time bins, was fed as the input.

### Complexity of the natural scenes

Natural movies have different scene contexts, which can be described in the pixel space on spatial and temporal scales. To characterize the spatiotemporal complexities of the scenes, first, each frame of the movie is sliced into patches of equal size, and the similarity between each patch and its neighboring patches is computed. For spatial complexity, we first calculate the SSIM between a patch and the eight neighboring patches in each movie frame. Next, we average the SSIM values across the neighboring patches. By averaging this value across all frames, we take the spatial correlation of the patch; the spatial complexity of the patch is 1 minus this value, i.e., a higher correlation means that the patch has a lower complexity. For example, for patch i (as shown in Figure S2), the spatial complexity *SC* is:

(Equation 9) $$SC_{i}=1-\frac{1}{T}\sum_{t=1}^{T}\frac{1}{n}\sum_{j\in neig\left(i\right)}^{n}SSIM\left(p_{i}^{t},p_{j}^{t}\right)\text{,}$$

where T is the number of frames in the movie, n is the patch number of j, which specifies its location as one of the eight neighbors of patch i, and p denotes the slice patch.

The calculation for the temporal complexity is similar; however, instead of comparing the patches in the same frame, the SSIM is calculated between a patch in frame t and its eight neighboring patches in the corresponding positions in frame t + 1, as well as between the patch at time t and the same patch at time t + 1. The temporal complexity of the patch is then obtained by performing similar steps as described for the spatial complexity. The formula for temporal complexity TC of patch i is as follows:

(Equation 10) $$TC_{i}=1-\frac{1}{T-1}\overset{T-1}{\underset{t=1}{\sum }}\frac{1}{n+1}\left(\overset{n}{\underset{j\in neig\left(i\right)}{\sum }}SSIM\left(p_{i}^{t},p_{j}^{t+1}\right)+SSIM\left(p_{i}^{t},p_{i}^{t+1}\right)\right)\text{.}$$

Using a patch size of 18 × 18 pixels, space and time complexities of both movies are shown in Figure 3D. Such complexities can affect the performance of the encoding models. To relate the performance of each model RGC to the complexity of an individual movie patch, we overlap the RF of each RGC with each patch, collocate those RGCs in that patch, and average all the CCs of the individual RGCs as the performance of the CNN and CRNN models for that particular image patch. Finally, we obtain a relationship between the complexity of each movie and the performance of each model, as shown in Figure 3E.

## Supplemental information

Document S1. Figures S1–S4 and Table S1

Document S2. Article plus supplemental information
