# Supplementary material for: Unraveling neural coding of dynamic natural visual scenes via convolutional recurrent neural networks
Source: Patterns (N Y). 2021 Sep 17;2(10):100350. doi: 10.1016/j.patter.2021.100350 (PMC8515013; doi:10.1016/j.patter.2021.100350)
Supplement: Document S1. Figures S1–S4 and Table S1 [file mmc1.pdf]

Patterns, Volume *2*

Supplemental information

Unraveling neural coding of dynamic natural visual scenes via convolutional recurrent neural networks

Yajing Zheng, Shanshan Jia, Zhaofei Yu, Jian K. Liu, and Tiejun Huang

Supplementary Material:
Unravelling neural coding of dynamic natural visual scenes via convolutional recurrent neural networks

TABLE S1

NUMBER OF PARAMETERS IN THE DIFFERENT MODELS OF FIGURE. 6

**Stimulus**	**CNN**	**CRNN-32**	**CRNN-64**	**CRNN-128**	**CRNN-256**
movie 1	2470×10^5	55×10^5	84×10^5	142×10^5	260×10^5
movie 2	1129×10^5	41×10^5	56×10^5	87×10^5	150×10^5

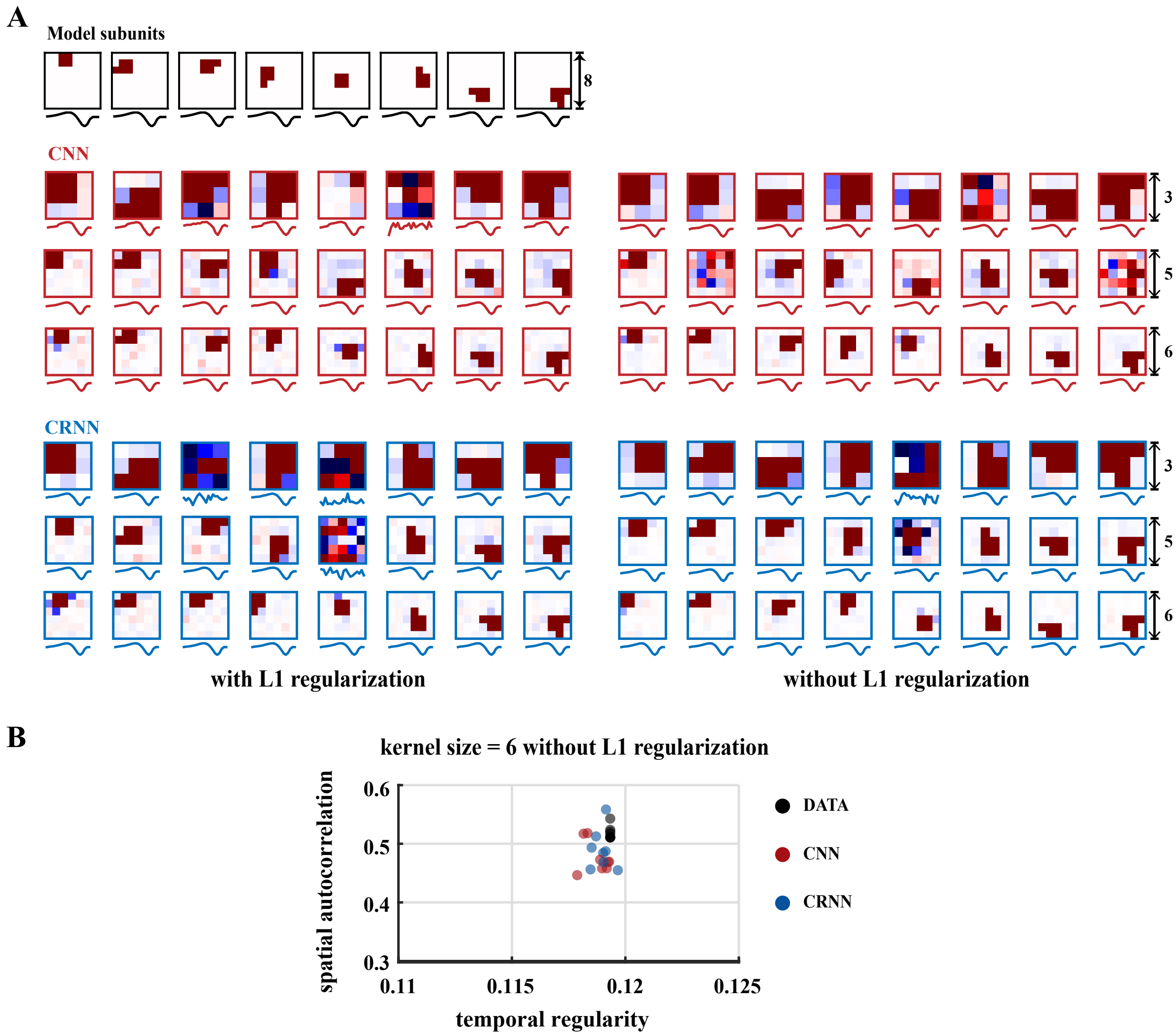

Figure. S1. Related to Figure. 2. CNN and CRNN models with different settings. **A.** Visualization of the convolutional filters learned in the CNNs and CRNNs with different kernel sizes of 3, 5 and 6, with and without ℓ_1 regularization. **B.** The spatial autocorrelation and temporal regularity of the models with conv1 kernel size equal to 6 without ℓ_1 regularization.

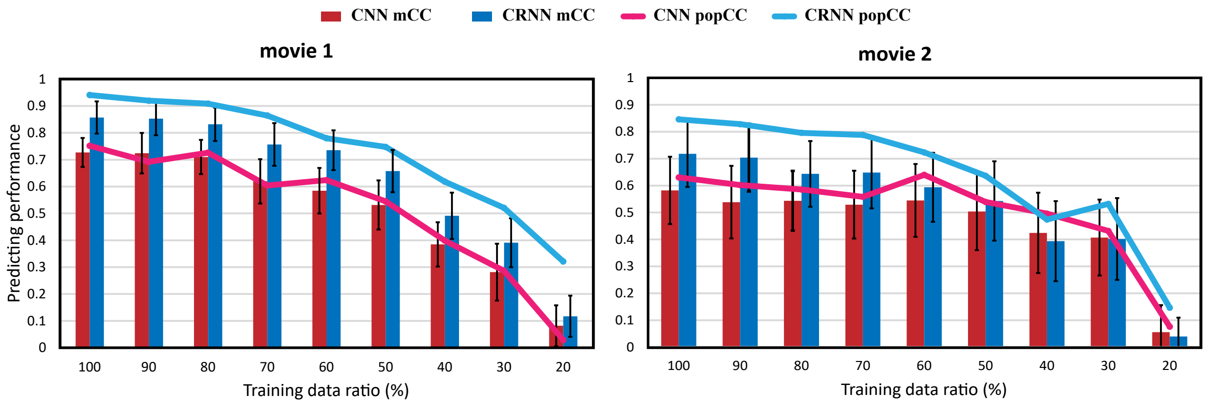

Figure. S2. Effect of the size of training data on the models. The original sample size ratio of the training data and testing data is 1:1. The size of the training data is changed from 20% to 100%. Both CNN and CRNN can maintain the predicting performance with only 70% training data. Surprisingly, with only about 30% of data, CRNN and CNN models can still obtain some level of performance (mCC: 0.39 v.s. 0.28 on movie 1, 0.40 v.s. 0.41 on movie 2; pCC: 0.52 v.s. 0.29 on movie 1, 0.53 v.s. 0.43 on movie 2). mCC refers to the average value of CC between the response of RGCs and the output of the models; popCC represents the CC between the response of population RGCs and the average output of the models.

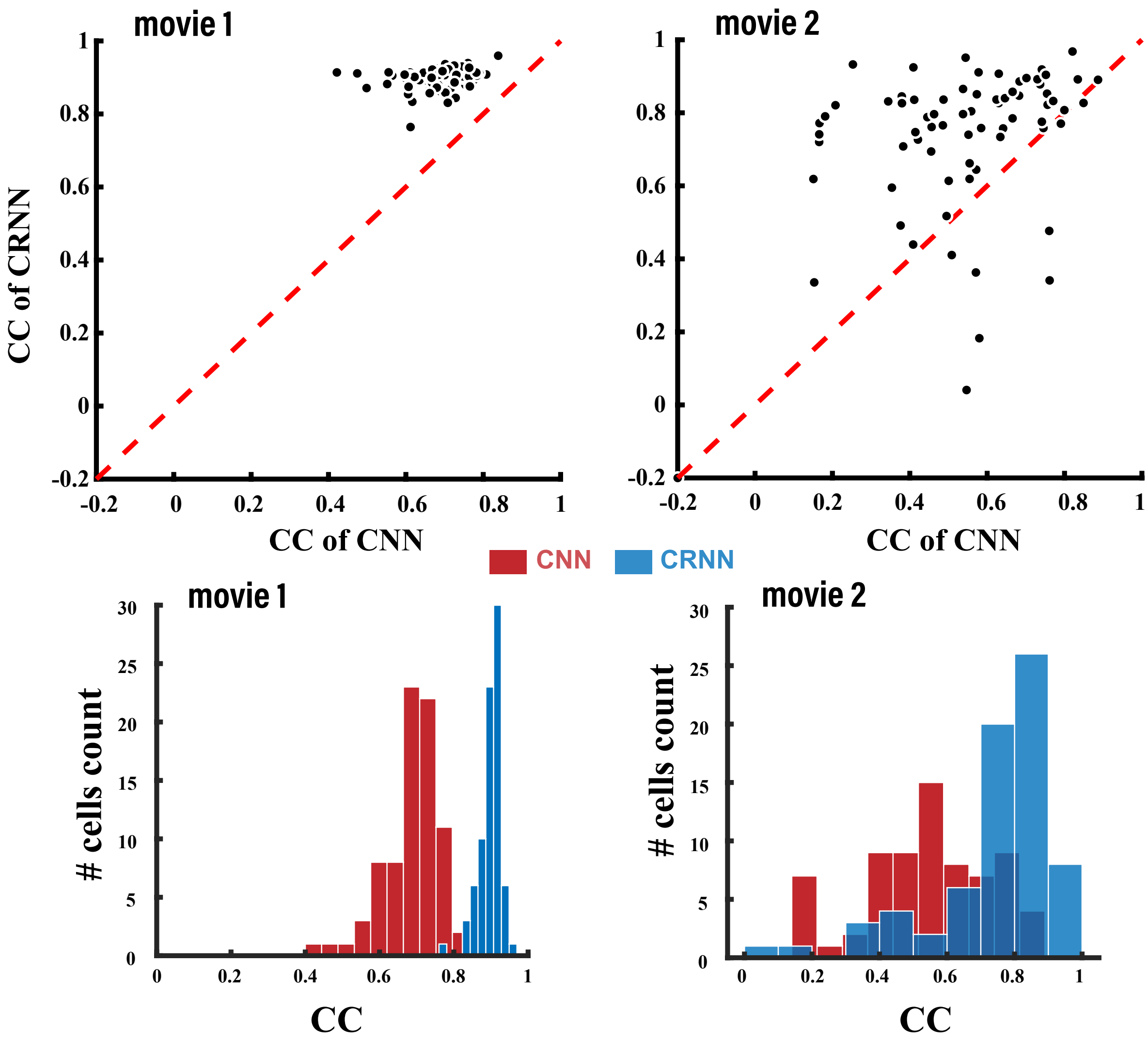

Figure. S3. Performance of the models trained with single trial spike trains. Instead of using the trial-averaged firing rate as neural response, one can use single trial spike train. Here we use one random trial as data and compute the correlation coefficient (CC) between the data and the model outputs from both CNN and CRNN for both movies.

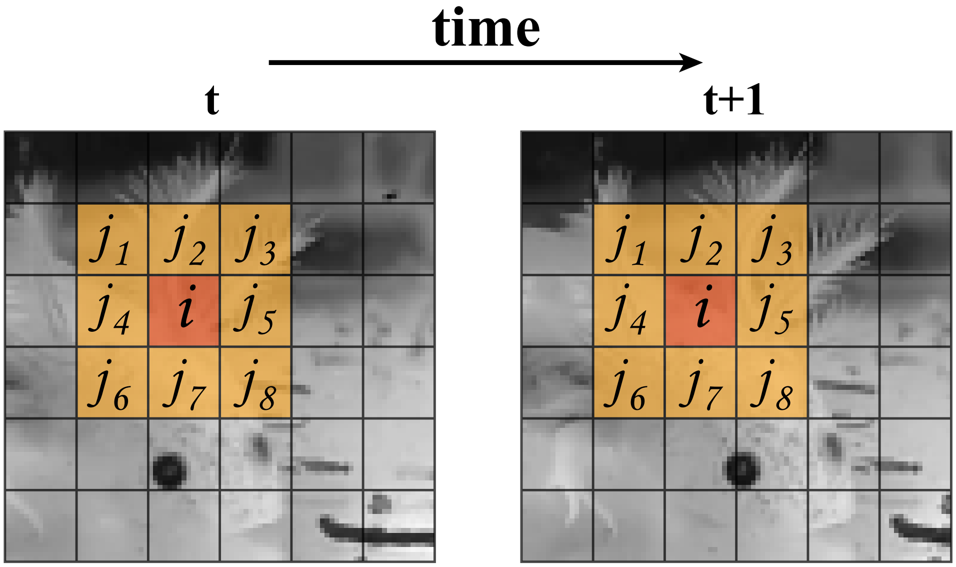

Figure. S4. Measurement of the complexity of dynamic visual scenes. First, the movie frame is sliced into patches of equal size, and the similarity between each patch is computed with its neighbouring patches. The structural similarity index (SSIM) between each patch and its eight neighbouring patches in each movie frame is calculated. The mean value of all the frames is calculated as the spatial correlation of the patch. The complexity is inversely proportional to the correlation with a higher value of correlation representing lower complexity of movies. For example, for the patch i shown in the Fig. S4, its spatial complexity SC is: $SC_i = 1 - \frac{1}{T}\sum_{t=1}^{T}\frac{1}{n}\sum_{j\in neig(i)}^{n} SSIM(\boldsymbol{p}_i^t, \boldsymbol{p}_j^t)$. Here T is the frame number of the movie, n is the patch number of j, which specifies its location as one of the eight neighbours of patch i, and $\boldsymbol{p}$ denotes the slice patch. In terms of time complexity, the operation method is similar. However, instead of comparing the patches in the same frame, the SSIM is calculated on the patches in the corresponding eight neighbours and the corresponding positions in the next frame. The time complexity of each patch is also obtained by taking the mean value of the frames of the image. The time complexity TC of patch i is calculated as: $TC_i = 1 - \frac{1}{T-1}\sum_{t=1}^{T-1}\frac{1}{n+1}\left(\sum_{j\in neig(i)}^{n} SSIM(\boldsymbol{p}_i^t, \boldsymbol{p}_j^{t+1}) + SSIM(\boldsymbol{p}_i^t, \boldsymbol{p}_i^{t+1})\right)$.
